# NEW PATHS TO NEUROTOLOGY IN BRAZIL

**DOI:** 10.1016/S1808-8694(15)30507-3

**Published:** 2015-10-18

**Authors:** Marco Aurélio Bottino, Maurício Malavasi Ganança, Roseli Saraiva Moreira Bittar Roseli Saraiva Moreira Bittar, Fernando Freitas Ganança, Mario Edvin Greters

**Keywords:** diagnosis, balance, dizziness, treatment, vertigo

In recent decades neurotology, as it happened to all medical fields, has gone through striking transformations. These transformations happened for numerous reasons, such as an increase in longevity, a search for quality of life, the development of new diagnostic investigation technologies and new treatment options. In parallel to that, conceptual models were reformed and the focus of neurotology shifted from the labyrinth to balance. Not only as a consequence of these changes, but also because of the high prevalence of patients complaining of dizziness in our medical offices, many other health care professionals started to diagnose and treat these body balance alterations. We understand that today, neurotology is a multidisciplinary specialty in which the otorhinolaryngologist has a predominant role to play, because he/she has the assessment, investigation and approach knowledge regarding the principal balance organ: the ear. It is paramount to champion our stake with professionalism and ethics.

Having in mind this new and important role played by otorhinolaryngology in the field of body balance, the ABORL-CCF chairman, Prof. Ricardo Ferreira Bento, suggested the setting up of a department to deal with the interests of otorhinolaryngologists in this field - the recently appointed Neurotology Committee of the ABORL-CCF. Its goals include: (1) standardization of diagnostic and treatment approaches; (2) quality research and teaching in this field; (3) disclosure of the progresses made in the different aspects of neurotology.

We feel honored for having been appointed to and we accept the invitation to organize this new ABORL-CCF department which now starts. We are at the brink of a new journey and we invite all our colleagues to participate, sending us suggestions and requests which may help us create a strong and representative department for Brazilian Neurotology.

